# Comparison Between Clinical and Pathological Staging After Elective Neck Dissection in Head and Neck Cancer

**DOI:** 10.7759/cureus.40881

**Published:** 2023-06-24

**Authors:** Mafalda Martins Sousa, Joana Guimarães, Eurico Monteiro

**Affiliations:** 1 Otolaryngology - Head and Neck Surgery, Centro Hospitalar Universitário de São João, Porto, PRT; 2 Otolaryngology - Head and Neck Surgery, Instituto Português de Oncologia do Porto, Porto, PRT

**Keywords:** pathological staging, head and neck cancer pathology, squamous cell carcinoma (scc), elective neck dissection, clinical staging

## Abstract

Introduction: Head and neck squamous cell carcinomas (HNSCC) are the most common malignancies in the head and neck. Previous studies have shown discrepancies in clinical and pathological staging, with a significant number of head and neck cancer patients who were not correctly staged. This has important implications regarding the treatment and prognosis of these patients. The aim of this study was to analyze potential disagreements in clinical and pathological staging in patients with head and neck cancer who underwent elective neck dissection.

Methods: A retrospective study of patients with squamous cell carcinoma of the head and neck, who underwent elective neck dissection, between January 2018 and December 2020.

Results: We analyzed 87 patients, with an average age of 64 ± 10.05 years, of whom 96.6% were male. The primary tumor location was the glottis (31%), oropharynx (26.4%), hypopharynx (19.5%), supraglottis (11.5%), and oral cavity (11.5%). In 87.3% there was a history of smoking and/or drinking. Pathological N (pN) staging was higher than clinical staging in 34.3% of patients (N1 in 22.9%; N2 in 8%; N3 in 3.4%). There were no significant differences between the number of nodes removed and the pN staging. However, there was a significant survival difference in patients with>15 nodes removed (p=0.05). There was also a significant difference in patients with pN up-staging regarding survival (p=0.005). Pathological T staging was different from clinical T staging (p<0.05), with an up-staging in 18.4% of the patients and a down-staging in 14.9%, without significant differences regarding survival or recurrence (p>0.05). Adjuvant treatment with radiotherapy was performed in 41.4% and with chemo-radiotherapy in 13.8% of the patients. Locoregional recurrence occurred in 17.5%.

Conclusion: This study revealed that clinical and pathological N staging after elective neck dissection disagreed in a substantial number of patients, with pathological upstaging and significant differences regarding survival. With relation to T staging, there were no significant differences regarding survival. We should be aware of staging disagreements since they can have significant implications on the treatment and prognosis of cancer patients.

## Introduction

Head and neck squamous cell carcinomas (HNSCC) mostly arise from mucosal epithelium in the oral cavity, pharynx, and larynx and are the most common malignancies in the head and neck [1]. Tobacco and alcohol consumption are the main risk factors in developing HNSCC and the combined misuse of both substances potentiates this risk. Rising numbers of oropharyngeal squamous cell carcinoma in the United States and Western Europe are also attributed to infection with human papillomavirus (HPV) [2]. Although there have been remarkable changes in diagnostic and therapeutic options, survival rates have not improved over the last three decades. A major keystone in the treatment selection and prognosis assessment is the analysis and classification of the cervical lymph node status. Therefore, an accurate evaluation of the neck lymph nodes is of great importance [3]. The tumor, node, and metastasis (TNM) classification is the most reliable system for classifying the extent of the primary tumor and regional and distant metastases [4]. Head and neck cancer clinical staging relies on the clinical examination combined with CT, MRI, or positron emission tomography (PET) [5-6]. Pathological staging is based on the surgical removal of tissue and detailed histopathological analysis [7]. At the time of diagnosis, treatment strategies are largely based on clinical staging. However, previous studies have shown discrepancies between clinical and pathological staging, revealing that a significant number of head and neck cancer patients are not correctly staged [5, 8-9]. Therefore, the purpose of this study was to compare clinical and pathologic staging in head and neck patients who underwent elective neck dissection. To our knowledge, this is the first study to analyze exclusively patients with HNSCC who had no lymph node metastasis in clinical staging (cN0).

## Materials and methods

We performed an observational retrospective study of patients with HNSCC, who underwent elective neck dissection, between January 1, 2018 and December 31, 2020, in the Otorhinolaryngology department of an oncological tertiary center. The study protocol was approved by the Medical Ethics Committee and was carried out in accordance with the principles of the Declarations of Helsinki. Patients without the essential clinical or pathological staging data were excluded. Demographic and clinical data were collected by analyzing patients’ medical records. The 8th edition of the American Joint Committee on Cancer (AJCC) staging system was used for tumor staging [7]. Clinical staging was performed by analyzing medical records and interpreting radiological reports of pre-operative imaging exams, namely CT scans, MRI, and PET-CT, and pathological staging was done via the interpretation of histopathological reports. A descriptive analysis of the patients’ characteristics was performed, taking into consideration absolute and relative frequencies for categorical variables and the mean and standard deviation for continuous variables. The normality of continuous variables was assessed with the Kolmogorov-Smirnov test. To determine an association between categorical variables, we used the chi-square test. To assess the accuracy of clinical and pathological staging, the Cohen Kappa coefficient was estimated. Survival curves were made using the Kaplan-Meier method, and differences were examined with the log-rank test. All statistical analyses were made with the software IBM® SPSS® Statistics version 27 (IBM Corp., Armonk, NY), and p-values < 0.05 were considered statistically significant. 

## Results

The study population included a total of 87 Caucasian patients (84 males and 3 females) with HNSCC, with a mean age of 64 ± 10.05 years old. The primary tumor was located in the glottic region in 31.03% of the patients, the oropharynx in 26.44%, the hypopharynx in 19.54%, the supraglottic region in 11.49%, and the oral cavity in 11.49%. Most of the patients (56.3%) had both smoking and drinking habits, 27.6% smoked without drinking habits, and 3.4% had drinking habits but denied ever smoking (Table 1).

**Table 1:** Demographic and clinical characteristics of the study population.

Age (mean +/- SD)	64 ± 10.05
Gender [n (%)]	
Male	84 (96.6)
Female	3 (3.4)
Primary tumor site [n (%)]	
Glottis	27 (31.03)
Oropharynx	23 (26.44)
Hypopharynx	17 (19.54)
Supraglottic	10 (11.49)
Oral cavity	10 (11.49)
Smoking [n (%)]	24 (27.6)
Drinking [n (%)]	3 (3.4)
Smoking + drinking [n (%)]	49 (56.3)
Neck dissection [n (%)]	
II-IV	69 (79.3)
II-V	10 (11.5)
I-III	8 (9.2)
Number of nodes removed [n (%)]	
15	77 (88.5%)
<15	10 (11.5)

All the patients included were cN0 and cM0. The time between clinical staging and elective neck dissection was on average 1.8 months (1-3 months). Selective neck dissection was performed, including levels II-IV in 69 (79.3%) patients, levels II-IV in 10 (11.5%) patients, and levels I-III in 8 (9.2%) patients, depending on the location of the primary tumor. Regarding the number of nodes removed during elective neck dissection, we established two groups (< 15 and 15 nodes on each side). In 77 (88.5%) patients, 15 nodes were removed (Table 1). There were no significant differences between the number of nodes removed and the Np staging (qui-square, p = 0.426). Only 57 (65.5%) patients judged to be clinically N0 were found to be N0 pathologically. In the remaining patients, there was an upstaging: 20 (23%) patients were pN1, 7 (8%) were pN2, and 3 (5.7%) were pN3 (Table 2). 

**Table 2:** Pathological N staging.

pN staging	n (%)
pN0	57 (65.5)
pN1	20 (23)
pN2	7 (8)
pN3	3 (3.5)

The agreement between clinical and pathological T stages was moderate (Cohen’s Kappa: 0.501; p < 0.001), with an upstaging in 9.1% and a downstaging in 8% of the patients. Regarding cT, most of the patients (55.2%) were cT4, 34.5% were cT3, and 10.3% were cT2 (Tables 3-4).

**Table 3:** Clinical T staging.

cT staging	n (%)
cT2	9 (10.3)
cT3	30 (34.5)
cT4	48 (55.2)

**Table 4:** Pathological T staging.

pT staging	n (%)
pT2	8 (9.2)
pT3	31 (35.6)
pT4	48 (55.2)

With pathological T staging, 55.2% were pT4, 34.5% were pT3, and 10.3% were pT2. Adjuvant treatment with radiotherapy was performed in 41.1% of patients and with chemoradiotherapy in 13.8%. Locoregional recurrence occurred in 17.5% of patients. 

Survival outcomes

**Figure 1:**
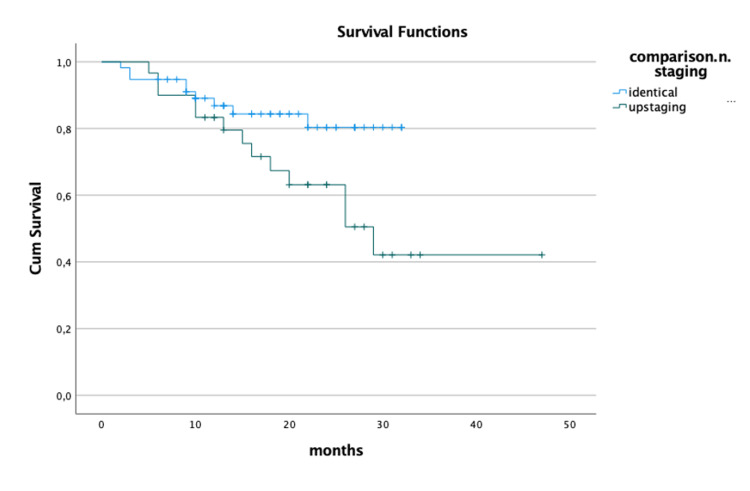
Survival Kaplan-Meier curves regarding N staging.

Among the 87 patients who underwent surgery, 22 (25.3%) died, with a mean time to death of 12.91 ± 7.9 months. Among surviving patients, the median follow-up in Otorhinolaryngology consultation was 20.34 ± 8.4 months. Disease recurrence was observed in 17.5% of patients, of whom 26.7% are still alive. Using Kaplan-Meier curves, we found a significant difference in the 5-year survival rate in patients with pathological N upstaging (log-rank test, p = 0.04; Figure 1). 

**Figure 2:**
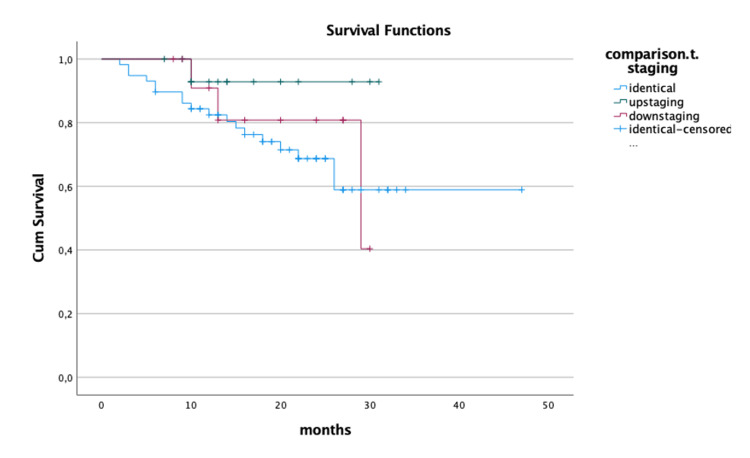
Survival Kaplan-Meier curves regarding T staging.

There were no significant differences regarding survival between patients with identical, upstaging, or downstaging on pathological T (log-rank test, p = 0.29; Figure 2). 

## Discussion

Age and gender distribution as well as high tobacco and alcohol consumption in this study are in accordance with previous studies [4, 8-10]. The main purpose of this study was to compare clinical and pathological staging in patients with head and neck cancer who underwent elective neck dissection. With regard to N staging, there was an upstaging in 34.5% of the patients. For T staging, there was moderate agreement, with differences in 17.1% of the patients. Of notice, physical examinations were performed by senior ear, nose, and throat (ENT) surgeons, who specialized in head and neck oncology, and clinical staging was complemented with high-resolution imaging examinations (CT, MRI, and/or PET). Previous studies have compared clinical and pathological staging agreements; however, our study was applied only to patients who were clinically N0, since we aimed to study occult lymph node metastasis and the impact they have on the treatment and prognosis of the patients. For instance, regarding oral cavity cancers, disagreements on T staging ranged from 12.7% to 55.9% and on N staging from 17.5% to 69.5% [10-13]. Celakovsky et al. found disparity in T staging in 13.5% of glottic cancers and 7.4% of supraglottic tumors, and differences in N staging in 40.7% of glottic cancers and 11.4% of supraglottic tumors [4]. Staging disagreements have major implications regarding the treatment of oncological patients since downstaged patients can have their treatment de-escalated to reduce unnecessary treatment morbidities, and patients with an upstage can be proposed to more appropriate adjuvant treatments. Regarding clinical staging, tumor dimensions and node size determined by manual palpation on physical examination are relatively inaccurate [9, 14]. The lower limit for node palpation has been reported to be 0.5 cm in superficial areas and 1 cm in deeper regions. A CT scan is used to improve accuracy, but it is inappropriate for detecting micrometastases [9, 14-15]. As a result, microscopic deposits and extracapsular spread may not be clinically detected and can only be accurately identified by pathological examinations [14]. In our study, there were no significant differences regarding the number of nodes removed during elective neck dissection and pN staging. This is probably due to the fact that most of the patients had a high number of nodes removed. We found a significant difference regarding survival in patients with pathological N upstaging, but there were no significant differences in survival in disagreements between clinical and pathological T staging. Other retrospective studies have shown increased mortality for pathological T upstaging [4], N upstaging [4], or both [11]. Previous reports have also shown no differences in mortality or disease-free survival between groups [8-9, 12-13]. Treatment modifications according to pathological staging may justify these findings. Studies are still lacking to determine the role of staging discrepancies in overall and disease-free survival. This study has some limitations. It is a retrospective study that included patients who underwent elective neck dissection in the same institution, but clinical staging and surgeries were performed by different head and neck surgeons, and specimens were analyzed by different pathologists. Additionally, the small follow-up period was a limitation for survival analysis and a larger sample might potentially alter the conclusions of this study. Nonetheless, to the best of our knowledge, this is the first study to access only patients with head and neck cancer who were clinically N0 and underwent elective neck dissection. Our population is from a single institution with standardized protocols that are crucial to the reliability of our results.

## Conclusions

In patients with head and neck cancer undergoing elective neck dissection, clinical and pathological N staging after elective neck dissection disagreed in an important number of patients, with pathological upstaging and significant differences regarding survival. Regarding T staging, there was a moderate agreement with no significant differences regarding survival. In this study, the time between clinical staging and surgery was on average 1.8 months. Of notice, all patients underwent elective neck dissection in the same institution, but clinical staging and surgeries were performed by different head and neck surgeons, and specimens were analyzed by different pathologists. Staging disagreements can have important implications for oncological practice, so it is crucial to acknowledge them. We should be aware that thorough elective neck dissection is of great importance since it can modify the treatment and prognosis of patients with HNSCC, who had no signs of lymph node metastasis in clinical staging.
